# A new species of *Tridactylogonus* Jeekel, 1982 from South Australia (Diplopoda, Polydesmida, Paradoxosomatidae)

**DOI:** 10.3897/zookeys.703.20986

**Published:** 2017-09-28

**Authors:** Robert Mesibov

**Affiliations:** 1 West Ulverstone, Tasmania 7315, Australia

**Keywords:** Diplopoda, Polydesmida, Paradoxosomatidae, South Australia, Australia

## Abstract

*Tridactylogonus warrenbenensis* **sp. n.** is described from Warrenben Conservation Park at the southern end of the Yorke Peninsula in South Australia. Like *T. obscurus* Jeekel, 1982 and *T. rugosissimus* Jeekel, 2002, the new species has prominent cellular sculpturing on the prozonites and granulose sculpturing on parts of the metazonites. Unlike its congeners and most species in the subfamily Australiosomatinae, the new species lacks a femoral process or tubercle on male leg 1.

## Introduction

**Figure 1.**
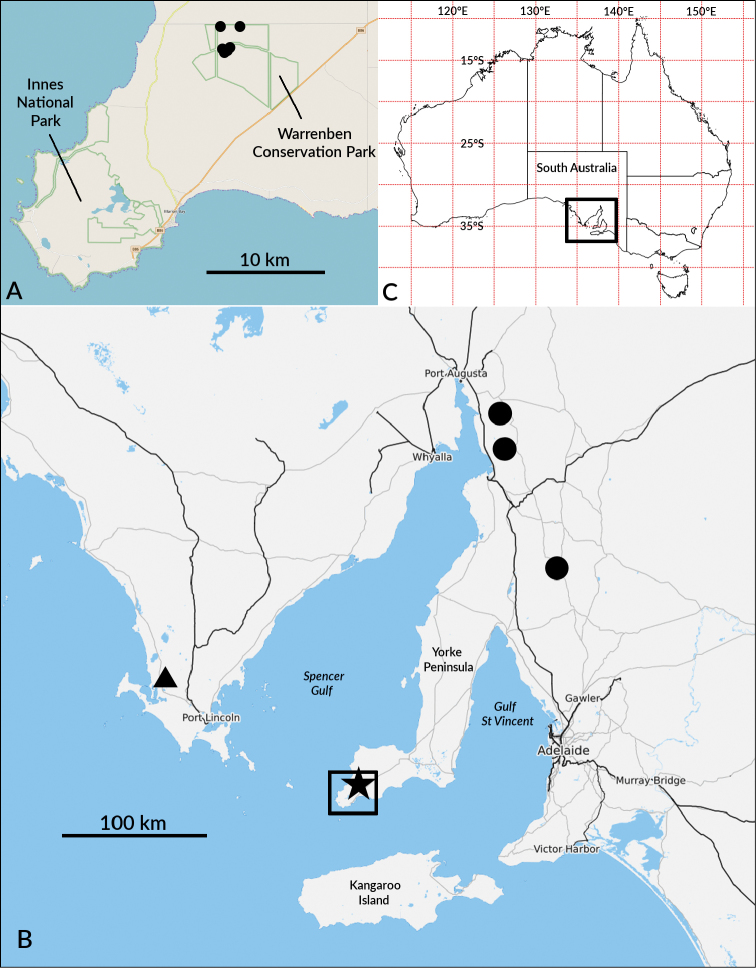
Known distribution of *Tridactylogonus* species as of 1 September 2017. **A** Localities for *T. warrenbenensis* sp. n. (filled circles) in Warrenben Conservation Park **B** Localities for *T. obscurus* Jeekel, 1982 (filled circles), *T. rugosissimus* Jeekel, 2002 (triangle) and *T. warrenbenensis* sp. n. (star); rectangle on Yorke Peninsula shows extent of map **A**. **C** Map of Australia; rectangle in South Australia shows extent of map **B**. Locality data for *T. obscurus* and *T. rugosissimus* from Mesibov (2006–2017); base maps for maps **A** and **B** from Open Street Map project (https://www.openstreetmap.org).

I collected the new species described here during two recent visits to the lower Yorke Peninsula in South Australia (Fig. 1). It was the only native polydesmidan species I found in the area, much of which has been heavily colonised by the introduced Portugese millipede *Ommatoiulus moreleti* (Lucas, 1860). It is a remarkably atypical species within the Australian Paradoxosomatidae, as it lacks a femoral process on male leg 1 and has prominent cuticular sculpturing on the prozonites and metazonites.

## Materials and methods

All specimens are stored in ethanol in the South Australian Museum (SAM). Body measurements were estimated with a Nikon SMZ800 binocular dissecting microscope using an eyepiece scale. Stacks of colour images were manually generated using a Canon EOS 1000D digital SLR camera mounted on the Nikon SMZ800 fitted with a beam splitter, then focus-stacked with Zerene Stacker 1.04 software. One gonopod was cleared in 80% lactic acid, temporarily mounted in a 1:1 glycerol:water mixture and imaged using an eyepiece video camera mounted on an Amscope binocular microscope. Preliminary drawings were traced from printed copies of the images, then corrected by reference to the actual gonopod. Scanning electron microscope images were acquired digitally using a Hitachi SU-70; body parts were examined after air-drying and sputter-coating with platinum, and later returned to alcohol. Figures were composed using GIMP 2.8 software. Parts of the backgrounds of the colour photomicrographs have been edited to remove distracting highlights and artifacts.

Locality details are given with latitude and longitude in decimal degrees based on the WGS84 datum. The estimated uncertainty for a locality is the radius of a circle around the given position in metres. Abbreviations: SA = South Australia, Australia; SAM = South Australian Museum, South Australia, Australia.

## Results

### Order Polydesmida Pocock, 1887

#### Suborder Strongylosomatidea Brölemann, 1916

##### Family Paradoxosomatidae Daday, 1889

###### Subfamily Australiosomatinae Brölemann, 1916

####### Tribe Antichiropodini Brölemann, 1916

######## Genus Tridactylogonus

Taxon classificationAnimaliaDiplopodaParadoxosomatidae

Jeekel, 1982

Tridactylogonus : Jeekel 1982: 128; Shelley et al. 2000: 135; Nguyen and Sierwald 2013: 1160.

######### Type species.

*Tridactylogonus obscurus* Jeekel, 1982, by original designation.

######### Other assigned species.

*T. rugosissimus* Jeekel, 1982, *T. warrenbenensis* sp. n.

######## Tridactylogonus warrenbenensis sp. n.

Taxon classificationAnimaliaDiplopodaParadoxosomatidae

http://zoobank.org/535BAB9A-9AAC-4E74-AFED-6708BC8D301C

Figs 1 (maps), 2, 3, 4, 5C, 5E

######### Holotype.

Male, Warrenben Conservation Park, SA, -35.1102, 137.0222 ±25 m, 30 m a.s.l., 16 August 2017, R. Mesibov and C. Arnold, open she-oak woodland, SAM OM2184.

######### Paratypes.

3 males, 7 females, details as for holotype, SAM OM2185-OM2194.

######### Other material.

1 male, 2 females, 1 juvenile, Warrenben Conservation Park, SA, -35.0926, 137.0121 ±100 m, 20 m a.s.l., 3 June 2016, R. Mesibov and T. Moule, degraded she-oak woodland, SAM OM2169; 1 juvenile, same details but -35.0922, 137.0464 ±100 m, 40 m a.s.l., 4 June 2016, burned she-oak and eucalypt woodland, SAM (not registered); 1 male, 2 juveniles, same locality but -35.1113, 137.0184 ±25 m, 30 m a.s.l., 15 August 2017, R. Mesibov, eucalypt and tea tree woodland, SAM OM2195-OM2197; 1 juvenile, same details but -35.1125, 137.0152 ±25 m, tea tree copse, SAM OM2198; 1 juvenile, same details but -35.1107, 137.0122 ±25 m, degraded she-oak woodland, SAM OM2199.

######### Diagnosis.

Differs from *T. obscurus* in having variably rugose rather than smooth metatergites, and in the anteromedial process of the gonopod telopodite being flattened rather than lanceolate. Differs from *T. rugosissimus* in having one process extending from the base of the solenomere rather than two. Differs from both *T. obscurus* and *T. rugosissimus* in lacking a femoral process or tubercle on male leg 1.

######### Description.

**Figure 2.**
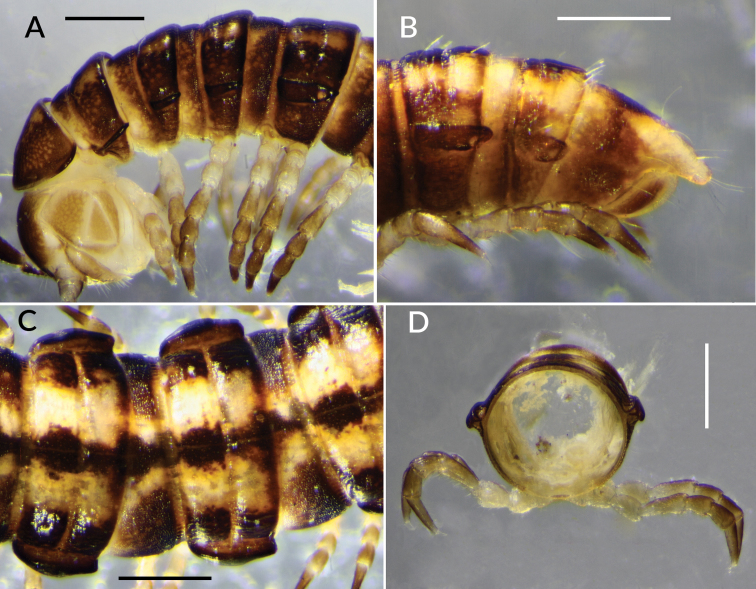
*Tridactylogonus warrenbenensis* sp. n. **A** Holotype (SAM OM2184), anterior end **B** Paratype male (ex SAM OM2185-OM2194), posterior end **C** Paratype female (ex SAM OM2185-OM2194), dorsal view of midbody rings **D** Paratype male (ex SAM OM2185-OM2194), posterior view of isolated midbody ring. Scale bars: 0.5 mm.

Male/female approximate measurements (all adults): length ca 12/14 mm, maximum midbody width 1.1/1.3 mm. Colour in alcohol (Fig. 2) light to medium brown, lightening ventrally, with yellowish paramedian bands dorsally, the bands on the prozonite closest together at the waist (Fig. 2C). Head yellowish laterally. Antennae brown, legs pale, in both cases darkening distally.

**Figure 3.**
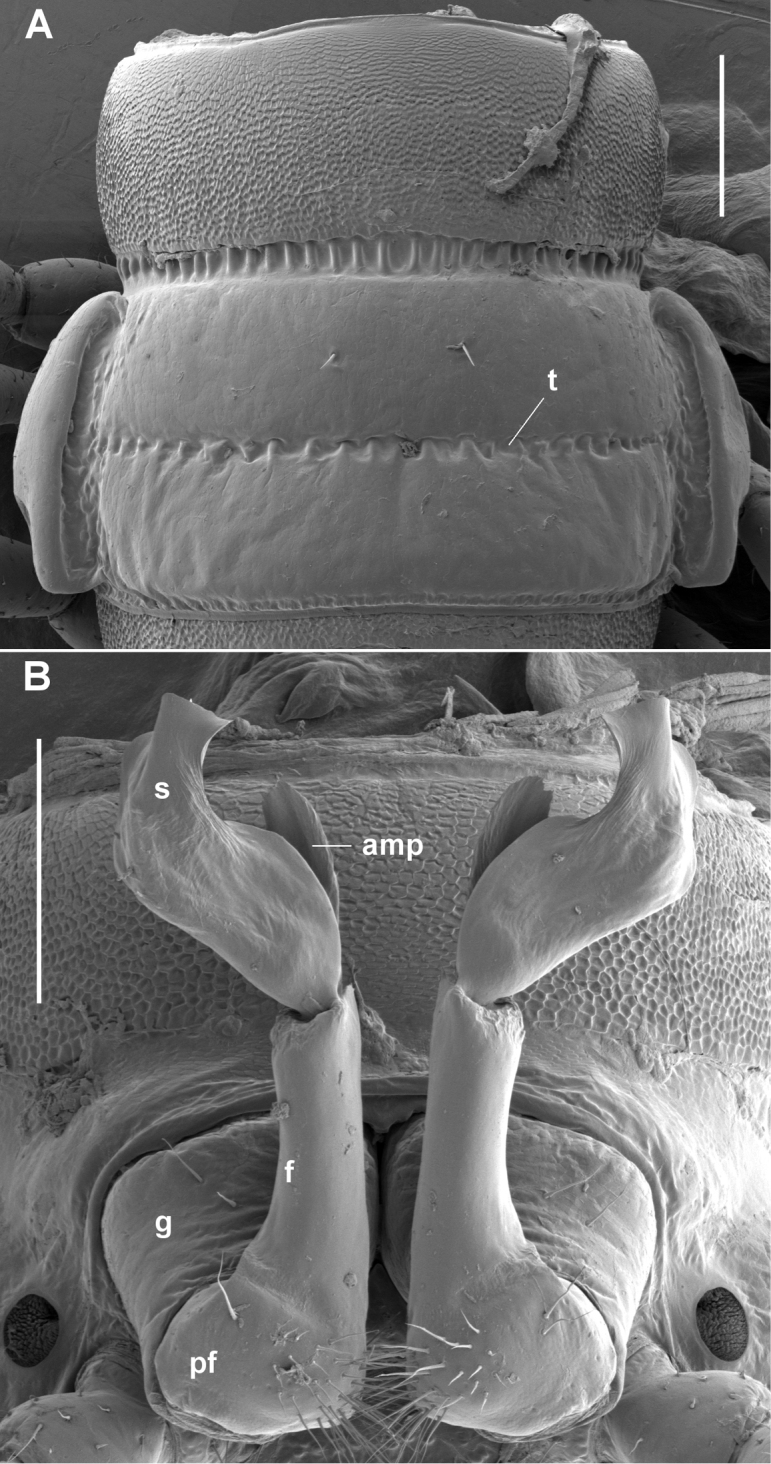
*Tridactylogonus warrenbenensis* sp. n., male (SAM OM2195). **A** Dorsal view of midbody ring; **t** = transverse furrow **B** Ventral view of gonopods in situ; **amp** = anteromedial process, **f** = femoral portion, **g** = gonocoxa, **pf** = prefemoral portion of telopodite, **s** = solenomere. Scale bars: 0.25 mm.

**Figure 4.**
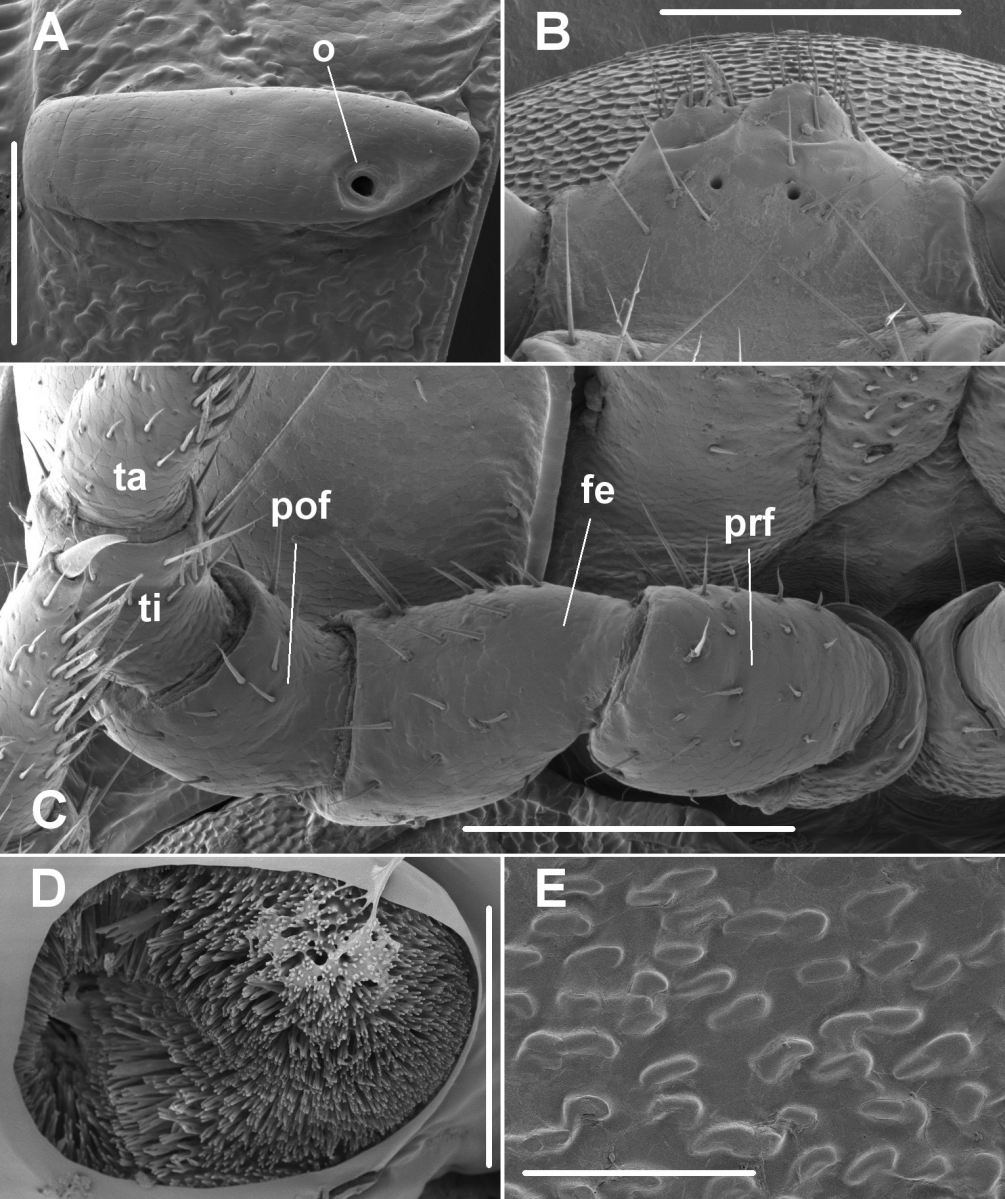
*Tridactylogonus warrenbenensis* sp. n., male (SAM OM2195). **A** Left lateral view of midbody paranotum; **o** = ozopore **B** Posterior view of sternal lamella between legs 5 **C** Posteroventral view of right leg 1; **fe** = femur, **pof** = postfemur, **prf** = prefemur, **ta** = tarsus, **ti** = tibia **D** Left lateral view of anterior spiracle on midbody ring **E** Close-up of microscopic ridges below paranotum in **A**. Scale bars: 0.2 mm (**A, B, C**); 0.05 mm (**D**), 0.1 mm (**E**).

Male with vertex bare, frons sparsely setose, clypeus moderately setose; vertigial sulcus distinct, ending just above level of antennal sockets; post-antennal groove shallow; antennal sockets separated by ca 1 socket diameter. Antennae clavate, reaching dorsally to rear of ring 2; antennomeres with relative lengths 6>(2=3)>(4=5); 6 thickest. Collum (Fig. 2A) half moon-shaped, strongly convex, rear margin straight, corners rounded and slightly upturned. Head slightly wider than collum; collum to ring 18 nearly uniform in width but rings 2 and 3 slightly narrower. Ring 2 paranotum (Fig. 2A) thin, dorsally concave, set lower than collum corner and ring 3 paranotum, extending slightly past posterior ring margin. Paranota on rings 3 and 4 (Fig. 2A) similar but thicker. Paranota on rings 5–18 (Figs 2A–D, 4A) prominent, set at ca 1/2 ring height; in lateral view rounded anteriorly, bluntly pointed posteriorly, extending just past posterior ring margin; dorsally concave medial to thickened lateral margin (Fig. 3A). No pleural keels on anterior rings.

Waist (Figs 2C, 3A) distinct, the zone between suture and anterior metazonite margin longitudinally ridged. Prozonites (Fig. 3) with prominent cellular sculpture dorsally, laterally and ventrally. Metatergites with transverse row of 4 prominent setae anterior to transverse furrow, the setae often abraded; posterior rings (Fig. 2B) with transverse row of setae near rear margin of metatergite. Metazonites laterally and ventrally with very small, irregular ridges (Fig. 4A, E) with flattened tops and rounded edges. Metatergites sometimes also with low, irregular folds, giving metatergite a variably wrinkled appearance (Figs 2D, 3A). Transverse furrow (Figs 2C, 3A) on rings 3–18 at ca 1/2 metatergite length, deeply impressed with some longitudinal ridging. Limbus a short, thin, uniformly wide sheet. Pore formula normal; ozopore (Fig. 4A) round, opening laterally at anterior end of narrow, ovoid depression at rear of paranotal margin. Spiracles on diplosegments above and just anterior to leg bases, rims thin; anterior spiracle (Fig. 4D) with rim slightly elevated, filter slightly emergent dorsally, filter elements flattened and with forked tips. Midbody sternites about as long as wide, sparsely and finely setose, cross impressions about equal in depth; no cones or other projections on any sternites. Midbody legs (Fig. 2D) with relative podomere lengths (femur=tarsus)>prefemur>(postfemur= tibia). Pre-anal ring (Fig. 2B) with a few long setae; epiproct extending well past anal valves, in dorsal view slightly tapering and truncate, tip ca 1/3 width of pre-anal ring; hypoproct paraboloid. Spinnerets in square array, dorsal setae longer than ventral, each pair set in narrow, shallow, transverse concavity on posterior surface of epiproct.

**Figure 5.**
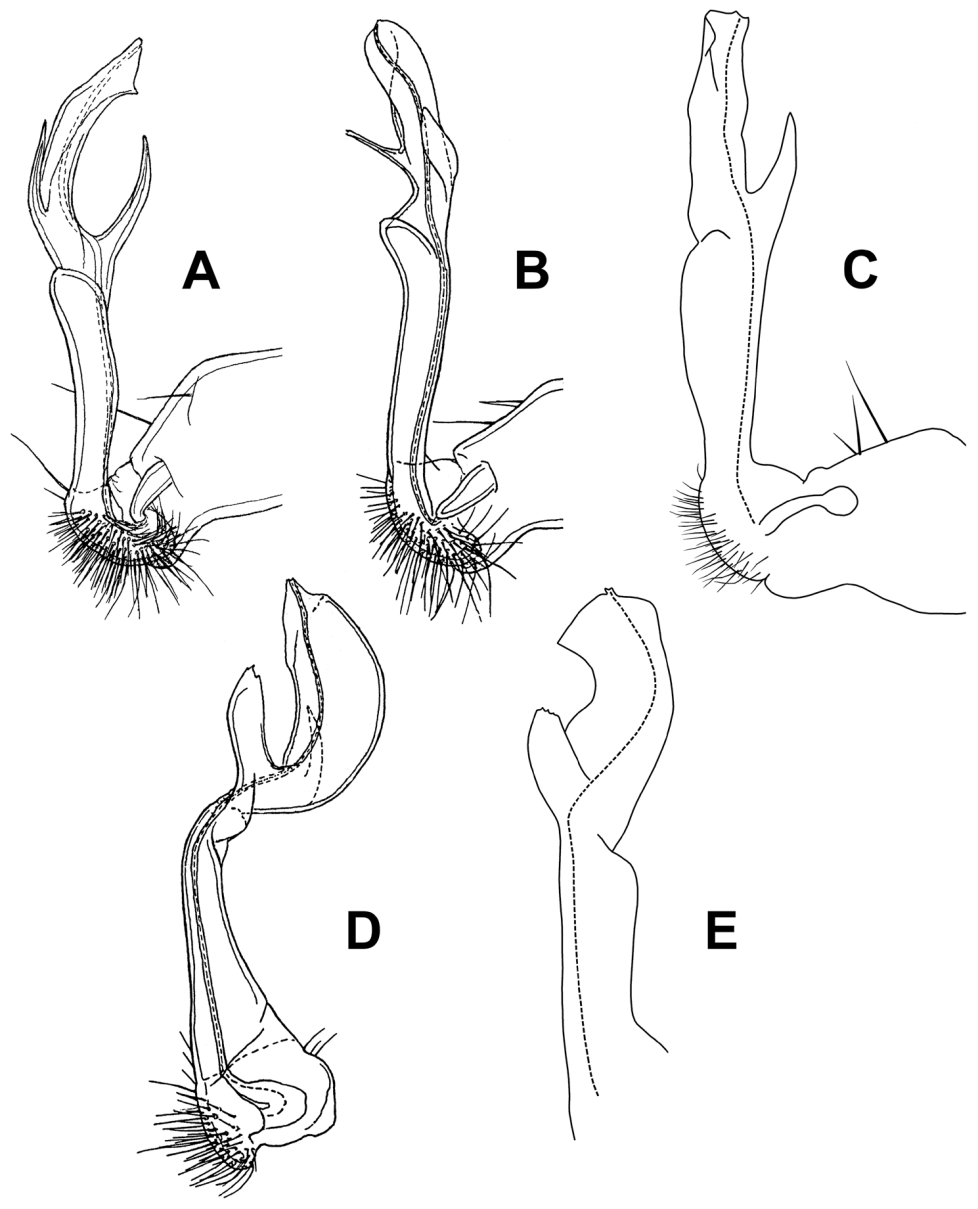
Gonopods of *Tridactylogonus* species. **A** *T. obscurus* holotype (after Jeekel 1982) **B, D** *T. rugosissimus* holotype (after Jeekel 2002) **C, E** *T. warrenbenensis* sp. n. paratype (ex SAM OM2185-OM2194) **A–C** medial views of right gonopod **D, E** anterior and anterior and slightly medial views, respectively **D** originally of left gonopod telopodite, here right-left reversed for comparison with right gonopod in **E**. Dashed line in **C** and **E** marks the prostatic groove. Drawings not to scale.

Leg 1 without ventral femoral process or tubercle (Fig. 4C). Gonopore small, round, opening on slight distomedial bulge of leg 2 coxa. Sternal lamella (Fig. 4B) between legs 5 ca 2/3 as wide as space between leg 5 coxae, short, distally with 2 bluntly rounded, setose projections. Sparse brush setae on leg 2 tarsus only. Anterior leg prefemora not swollen dorsally. Gonopod aperture (Fig. 3B) just wide enough to accommodate gonocoxae, ca 1/2 ring 7 prozonite width. Gonopod telopodites (Figs 3B, 5C, 5E) straight, parallel, reaching leg 6 bases when retracted; sternite between legpairs 6 and 7 slightly excavate. Gonocoxa short, truncate-conical, with a few long setae anterolaterally. Cannula prominent. Telopodite with prefemoral portion moderately setose medially, marked distally by very slight constriction and by obvious reduction in telopodite width. Femoral portion ca 1/2 acropodite length, subcylindrical, ending posteriorly in lip-like extension. Distal half of acropodite with two (not three) processes: broad, laminate, distally rounded-truncate solenomere, slightly concave anteriorly and broadly emarginate medially; and short anteromedial process, ca 1/2 solenomere length, directed slightly distomedially, laminate with rounded, sparsely microdentate distal margin. Prostatic groove prominent, running distally along anteromedial surface of femoral portion, then curving between bases of solenomere and anteromedial process and along anterior surface of solenomere, terminating as short, central projection on distal solenomere margin.

Female more robust than male; epigyne thickened but barely protruding; cyphopods not examined.

######### Name.

For the type locality, Warrenben Conservation Park.

######### Distribution.

Known from six localities over ca 4 km^2^ in Warrenben Conservation Park at the southern end of the Yorke Peninsula, South Australia (Fig. 1A, B). Found in bark litter under dead she-oak trees (*Allocasuarina stricta*) and in *Eucalyptus* sp. bark litter in shrubby, mallee-type vegetation on limestone at 20–30 m elevation. The area has an annual rainfall of ca 440 mm (Bureau of Meteorology 2017).

######### Remarks.

*Taxonomic affinities*. Although its gonopod telopodite is “bidactylous” rather than “tridactylous”, *T. warrenbenensis* sp. n. closely resembles the other two *Tridactylogonus* species in its small size and gonopod form. The genus was thought by Jeekel (1982, p. 128) to “stand rather isolated” within the tribe Antichiropodini, and its discovery “might seem to narrow the taxonomic disjunction between this tribe and the Australiosomatini”. However, as in other Australian Antichiropodini, especially *Aethalosoma* Jeekel, 2006, *Aulacoporus* Verhoeff, 1924, *Brochopeltis* Verhoeff, 1924, *Pseudostrongylosoma* Verhoeff, 1924 and *Walesoma* Verhoeff, 1928, the gonopod telopodite in *Tridactylogonus* has a narrow, straight femoral portion arising from a small, setose prefemoral portion, with the femoral portion clearly demarcated from the solenomere and any other apical processes. Also, as in *Aethalosoma*, *Dicranogonus* Jeekel, 1982, *Notodesmus* Chamberlin, 1920 and *Pogonosternum* Jeekel, 1965 (fig. 2 in Mesibov 2009, fig. 4B in Decker et al. 2017), the spiracular filter elements in the new species are flattened and have forked tips.

Within the genus, *T. warrenbenensis* sp. n. is most similar to *T. rugosissimus* in gonopod form and in metazonite sculpturing (see below), which Jeekel (2002, p. 65) thought could distinguish the latter species “from all other known Australian Paradoxosomatidae”. The new species is remarkable in lacking a femoral process or tubercle on male leg 1, a character almost universally present in Australiosomatinae. Jeekel reported that the leg 1 femur in *T. obscurus* had “the usual ventral tubercle” (Jeekel 1982, p.130), and in *T. rugosissimus* “a small ventral tubercle” (Jeekel 2002, p. 64). Unfortunately I have not been able to examine the type and only known specimen of *T. rugosissimus*; it was not deposited in the South Australian Museum as proposed (Jeekel 2002, p. 60) and has not been found among material in the late Dr Jeekel’s study collection in the Naturalis Biodiversity Center in Leyden, the Netherlands (K. van Dorp, in litt., 17 September 2014).

*Surface sculpture*. Jeekel (1982, p. 129) noted “a fine, but quite conspicuous cellular structure” on the prozonites and “subgranulose” sides in *T. obscurus*. In *T. rugosissimus* the prozonites were said to have either “pronounced reticulate structure or minute and regular granulation”, while the metatergites were “coarsely and densely rugose, with irregular longitudinal or somewhat oblique or undulate rugae”, and the sides “coarsely granulose to subrugulose” (Jeekel 2002, p. 63). Cellular prozonite sculpturing seems to be a constant character state in *T. warrenbenensis* sp. n. and closely resembles the prozonite sculpturing in another Australian paradoxosomatid, *Desmoxytoides hasenpuschorum* Mesibov, 2006 (fig. 5A in Mesibov 2009). “Rugose” sculpturing of the metazonites is more variable. It is always present on the sides as irregular microscopic ridges, but is variably prominent on the metatergites as coarse, narrow wrinkles. Each microscopic ridge appears to project from one cuticular scale, but ridges on adjoining scales sometimes appear to be confluent (Fig. 4E), in contrast to the discretely spaced projections seen in some other recently examined Polydesmida (Mesibov 2009, Akkari and Enghoff 2011).

*Biogeography and conservation*. The three *Tridactylogonus* species occur around Spencer and St Vincent Gulfs in southern South Australia (Fig. 1B) and form an allopatric species mosaic. *T. warrenbenensis* sp. n. is so far known only from Warrenben Conservation Park (Fig. 1A), which covers ca 4000 ha and is managed for nature conservation. Over several days of searching in 2016 and 2017 I was unable to find any native millipedes in the nearby and larger Innes National Park (Fig. 1A), despite the presence there of apparently suitable woodland and scrub habitats. The National Park instead has a dense and almost ubiquitous population of the introduced Portugese millipede *Ommatoiulus moreleti* (Lucas, 1860). *O. moreleti* is also the most common millipede elsewhere on the Yorke Peninsula in both partly natural and entirely agricultural habitats. If *O. moreleti* has displaced native millipedes in Innes National Park, it may displace *T. warrenbenensis* sp. n. in future in Warrenben CP, which is linked to Innes NP by several large blocks of privately owned bushland. During my 2016 and 2017 visits I saw no *O. moreleti* in Warrenben CP.

## Supplementary Material

XML Treatment for Tridactylogonus

XML Treatment for Tridactylogonus warrenbenensis
